# An Unusual Case of Nephrotic Range Proteinuria in a Short-Standing Type 1 Diabetic Patient with Newly Diagnosed Systemic Lupus Erythematosus: A Case Report and Literature Review

**DOI:** 10.3390/medsci12040074

**Published:** 2024-12-16

**Authors:** Marco Dominguez Davalos, José C. De La Flor, Carlos Bedia Castillo, Roxana Lipa Chancolla, Celia Rodríguez Tudero, Jacqueline Apaza, Rocío Zamora, Michael Cieza-Terrones

**Affiliations:** 1 Department of Nephrology, Hospital Cayetano Heredia, Lima 15002, Peru; marco.dominguez.d@upch.pe (M.D.D.); michael.cieza@upch.pe (M.C.-T.); 2 Faculty of Medicine, Peruana Cayetano Heredia University, Lima 15002, Peru; carlos.bedia@upch.pe; 3 Department of Nephrology, Hospital Central Defense Gomez Ulla, 280467 Madrid, Spain; 4 Faculty of Medicine, Alcala de Henares University, 28805 Madrid, Spain; 5 Department of Rheumatology, Hospital Cayetano Heredia, Lima 15002, Peru; 6 Anatomic Pathology Department, Instituto Nacional de Salud del Niño, Lima 15082, Peru; rlipa@insnb.gob.pe; 7 Department of Nephrology, Hospital Universitario de Salamanca, 37007 Salamanca, Spain; crodrigueztu@saludcastillayleon.es; 8 Department of Nephrology, Hospital Rey Juan Carlos, 28933 Madrid, Spain; jacqueline.apaza@hospitalreyjuancarlos.es; 9 Department of Nephrology, Hospital Universitario General Villalba, 28400 Madrid, Spain; rocio.zamora@hgvillalba.es

**Keywords:** lupus podocytopathy, systemic lupus erythematosus, nephrotic syndrome

## Abstract

Background: Lupus podocytopathy (LP) is a non-immune complex-mediated glomerular lesion in systemic lupus erythematosus (SLE), characterized by the diffuse effacement of podocyte processes without immune complex deposition or with only mesangial immune complex deposition. LP is a rare cause of nephrotic syndrome in SLE patients with implications for prognosis and treatment. Case Report: We present the case of a 28-year-old woman with a medical history of type 1 diabetes mellitus (T1DM) who presented with lower limb edema, dyspnea, hypercholesterolemia, with nephrotic range proteinuria, without acute kidney injury, and laboratory findings compatible with auto-immune hemolytic anemia. They had negative infectious serology, positive antinuclear antibody (ANA), and an eye fundus examination showing diabetic retinopathy. A biopsy was performed to define the etiology of the renal involvement, which was compatible with LP. Following immuno-suppressive and antiproteinuric therapy, the patient evolved with the complete remission of the nephrotic syndrome. Conclusions: Lupus podocytopathy is an infrequent anatomopathological entity, so this case is presented as the first reported in Peru, and a literature review is made.

## 1. Introduction

Systemic lupus erythematosus (SLE) is a chronic autoimmune disease with multisystem involvement and the presence of autoantibodies. The 2019 European League Against Rheumatism/American College EULAR/ACR classification criteria for SLE include the presence of positive antinuclear antibody (ANA) at least once as obligatory entry criterion, followed by additive weighted criteria grouped in seven clinical (constitutional, hematologic, neuropsychiatric, mucocutaneous, serosal, musculoskeletal, renal) and three immunological (antiphospholipid antibodies, complement proteins, SLE-specific antibodies) domains, and weighted from 2 to 10. Patients accumulating ≥ 10 points are classified as SLE [1]. In Peru, the prevalence of SLE is 50 per 100,000 thousand inhabitants [2]. Kidneys are among the organs most commonly affected by this disease: up to 60% of people with SLE may have renal involvement. Lupus nephritis (LN) occurs in approximately 50% of SLE patients and is the most frequent, but it is not the only cause of kidney damage in SLE [3]. In general, patients with SLE and associated nephrotic syndrome present proliferative LN (class III/IV) or membranous LN (class V) (with or without concomitant proliferative lesions) on renal biopsy (RB) [4]. However, in rare cases, it is presented by an anatomopathological entity called lupus podocytopathy (LP), which is not included in the six classes of the International Society of Nephrology/Renal Pathology Society (ISN/RPS). LP is a non-immune complex-mediated LN, which has RB findings of normal glomeruli or focal segmental glomerulosclerosis (FSGS) lesions (with or without mesangial proliferation) with light microscopy (LM); the absence of subepithelial or subendothelial deposits with immunofluorescence and electron microscopy (EM); and diffuse pedicellar effacement with EM [5]. LP affects approximately 1% of individuals with nephrotic proteinuria due to SLE [6], and its estimated prevalence is 0.4–1 case per 10,000 inhabitants [7].

LP can be classified as a minimal change disease (MCD) or FSGS, including the morphological subtypes of FSGS (unspecified variant, perihilar variant, cellular, spiking or collapsing), the latter group reportedly having a higher rate of falls and a lower response to treatment [8].

Most patients with LP usually respond to a short course of high-dose glucocorticoids. However, within the subtypes of LP, the FSGS forms are the ones that have a worse response to steroids than MCD. The collapsing variant of FSGS in LP is described as the most aggressive. In cases of LP due to FSGS, given the more severe phenotype and lower response rates, steroids associated with another immunosuppressive drug should be considered for induction and maintenance therapy [9].

We present a case of nephrotic syndrome in a young woman with concomitant diagnoses of type 1 diabetes mellitus (T1DM) and SLE, in whom RB was able to define the etiology of the proteinuria.

## 2. Case Presentation

A 28-year-old woman with a medical history of T1DM came to the emergency department in August 2023, complaining of lower limb edema and foamy urine for 6 weeks. The patient also reported generalized weakness, hyporexia, and dyspnea during usual exertion. The patient had been diagnosed with T1DM three years ago at our institution and was undergoing irregular treatment with insulin, without the controls of glycemia or glycosylated hemoglobin. On physical examination, vital signs were within normal limits. She was pale 2+/3+ and the lower limbs had edema 2+/4+ with bilateral fovea. There were no cases of purpuric lesions, arthritis, hepatosplenomegaly, or Raynaud’s phenomenon. The laboratory tests showed the following: severe macrocytic hyperchromic anemia, reticulocytes: 21.4%, peripheral lamina with spherocytes, polychromatophilia, normoblasts, and Coombs +++. Alongside this, a 43.2% occurrence of urea/creatinine dissociation relative to normal urea and creatinine levels. In addition, she presented with severe hypoalbuminemia, hypercholesterolemia, and proteinuria in the nephrotic range. Urinalysis showed sterile leukocyturia without hematuria. The serological tests were negative for hepatitis B, HCV, and HIV. She had mild C4 hypocomplementemia, positive ANA, and the rest of the autoimmune studies were normal. The fundus study revealed moderate diabetic retinopathy. The ultrasound showed kidneys of a normal size and adequate echogenicity. The rest of the blood tests are shown in Table 1.

**Figure 1:**
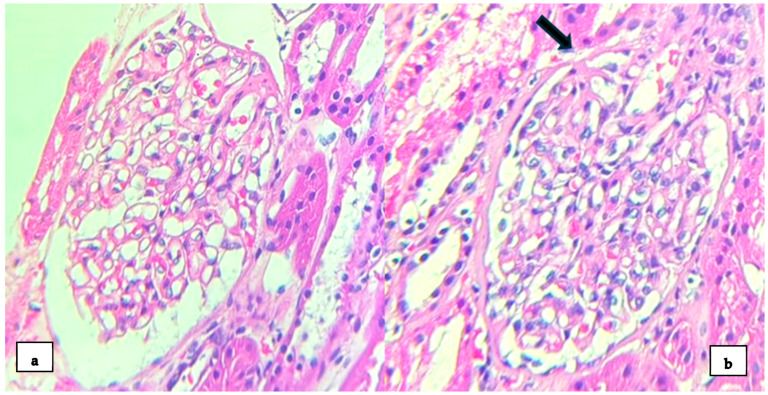
Kidney biopsy: (**a**) light microscopy shows the diffuse uniform thickening of the glomerular basement membrane (H-E stain × 400); (**b**) a glomerulus showing the adhesion of the glomerular basement membrane to Bowman’s capsule at the urinary pole (black arrow) (H-E stain × 400).

**Figure 2:**
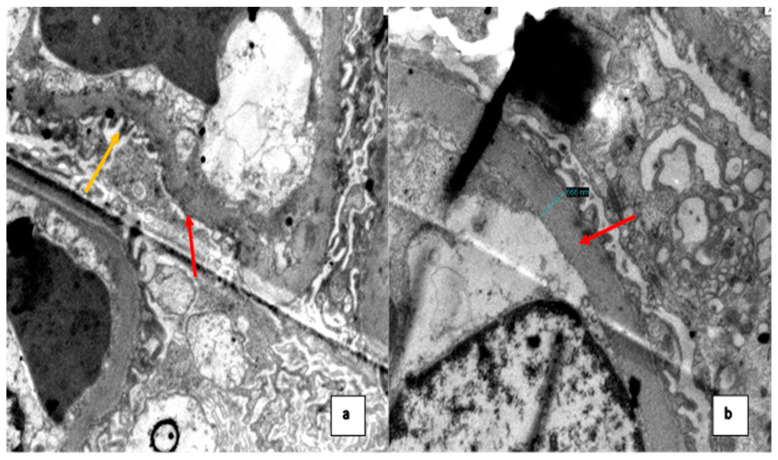
(**a**) Ultrastructural study shows pedicel effacement in most of the capillary perimeter (red arrow) and some segments with preserved pedicels (yellow arrow); (**b**) basement membrane with diffusely increased thickness (666 nm) (normal values for age and sex: 215–395 nm) (red arrow).

Due to the findings in the hematological analysis, the clinical diagnosis was autoimmune hemolytic anemia; methylprednisolone 250 mg intravenous (IV) for 4 days was indicated, with a subsequent control of hemoglobin (Hb) (8 g/dL). However, the patient presented with nephrotic syndrome with no clear etiology, so an RB was performed. LM showed 40 glomeruli, with one of them globally sclerosed. The remaining glomeruli show a diffuse uniform thickening of the glomerular basement membrane (GBM); no mesangial expansion was observed. In one of the glomeruli, the adhesion of the glomerular basement membrane to Bowman’s capsule at the urinary pole was observed (Figure 1). There were no signs of interstitial inflammation. Mild foci of interstitial fibrosis and tubular atrophy were observed. Under direct immunofluorescence (DIF), there was no significant staining for the IgG, IgA, IgM, C3, and kappa and lambda chains. EM showed diffuse pedicle effacement in 70% of the sample. Additionally, a diffusely thickened basement membrane was observed (Figure 2). There were no immune-type electron dense deposits in the mesangial regions, tubular basement membranes or capillary walls.

**Figure 3:**
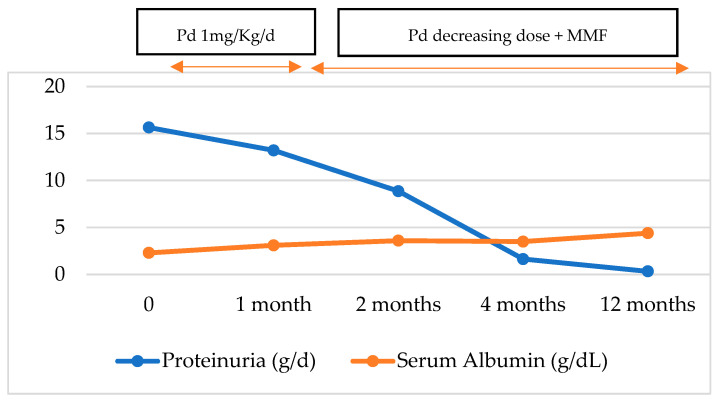
Evolution of proteinuria and serum albumin with the treatment. Pd: Prednisone; MMF: Mycophenolate Mofetil; g: gram; d: day.

Findings are compatible with a histological pattern of tip variant FSGS with the absence of subepithelial/endothelial deposits and diffuse podocytopathy, compatible with lupus podocytopathy. Findings of early diabetic nephropathy (DN) were simultaneously found. Rheumatology was consulted, and the patient was treated with furosemide 60 mg per day for 3 weeks, losartan 50 mg twice daily, and spironolactone 50 mg daily, which led to a decrease in edema. In addition, treatment was started with high-dose prednisone 60 mg/dL per day, hydroxychloroquine 400 mg daily, and mycophenolate mofetil (starting at 3 g/day and decreased to 2.5 g/day) to permit the tapering of corticosteroids, obtaining a complete response to the nephrotic syndrome. After one month follow-up in the clinic, the patient’s lower limb edema was resolved, proteinuria 24hr decreased to 13 g, 8.8 g, 1.64 g, and 0.34 g at 1, 2, 4 months, and 1 -year follow-ups, respectively (Figure 3).

## 3. Discussion

We present an unusual case of nephrotic syndrome in a patient with SLE and T1DM compatible with LP and early DN; this does not explain the picture of the patient’s nephrotic syndrome, and yet the good evolution after immunosuppressive treatment confirms the diagnosis of LP. To our knowledge, this is the first case report of LP in Peru and the second case in South America. The first was published by Pipino et al. [10] about a 28-years-old woman with nephrotic syndrome, as in our case. Kraft et al. reported in 2005 eight cases of patients with SLE, nephrotic syndrome, and RB findings of MCD, FSGS, or mesangial proliferative glomerulonephritis. The observation that the appearance of nephrotic syndrome frequently correlates with the appearance of systemic clinical features of SLE led to the idea that they are not two coexisting diseases. Therefore, podocytopathy is the result of active SLE, which gave rise to the term ‘lupus podocytopathy’ [11]. Hu et al. [9] described that nephrotic syndrome in LP frequently occurred as an initial symptom of SLE (88%). Our patient was diagnosed with nephrotic syndrome and hemolytic anemia as the initial clinical manifestation of SLE. Our patient was not diagnosed with malar rash, which is seen in 46% of patients with LP, nor arthritis, seen in 34% of cases. She did not present microscopic hematuria or acute kidney injury, which is consistent with the literature describing that microscopic hematuria and acute kidney injury are uncommon in LP, occurring in only 18% and 33% of cases, respectively. Moreover, anemia (immune component), low complement C4, and negative anti-double-stranded DNA (anti-dsDNA) were present in 26%, 28%, and 74% of cases, respectively [5].

We reported a patient with short-standing TD1M and LP. The coexistence of these entities in this patient raises intriguing questions about potential shared or synergistic pathogenic mechanisms. Both conditions involve immune-mediated damage to distinct cellular structures, podocytes in LP [8], and pancreatic beta cells in T1DM [12], but their intersection may provide insight into unique pathogenic interactions. Both SLE and T1DM involve dysregulated T-cell responses. In SLE, Th17 cells and imbalances in T-regulation promote inflammation and autoimmunity. Similarly, Th17-driven inflammation has been implicated in the destruction of beta cells in T1DM [13,14]. These shared cytokine pathways (e.g., IL-17, IL-2) could contribute to the overlap of renal damage in patients with both conditions. The poor glycemic control and diabetic retinopathy of this patient suggests chronic hyperglycemia, which may have initiated podocyte stress even before the onset of LP. The immune activation of lupus may have amplified this stress, leading to podocyte loss and proteinuria. EM findings in our patient indicated the thickening of the glomerular basement membranes, a distinctive feature of diabetic nephropathy. This structural change could make the glomeruli more vulnerable to the immune-mediated damage observed in LP.

With respect to global LP cases, Wang et al. reported a case series of thirteen patients with LP: eleven patients were female, ten had normal kidney function, seven had nephrotic syndrome and the absence of hematuria and six patients had anemia (it is not specified if it was autoimmune), like our patient. However, eleven patients had some positive value of the extractable nuclear antigen (ENA) profile, ten patients did not have a complete response, and nine patients showed MCD-type lesions, unlike our case [15]. Cobb et al. reported a case series of eighteen patients with LP; ten showed FSGS lesions, the initial average laboratorial findings were compatible nephrotic syndrome, and the patients displayed a good response, like our patient. Fourteen patients were treated with prednisone alone (one patient was treated with steroids plus mycophenolate mofetil) and the initial average creatinine was altered, unlike our case [16]. And finally, Salvatore et al. reported a case series of 19 patients with FSGS (collapsing glomerulopathy) and SLE: 15 patients were females, 17 patients had nephrotic syndrome, and 13 patients did not have hematuria like our case. Eleven patients had positive anti-dsDNA and seven of thirteen patients with follow-up data progressed to end-stage renal disease, unlike our case [17].

The most commonly used diagnostic criteria for LP are the following: (1) the clinical presentation of nephrotic syndrome in a patient with lupus, (2) normal glomeruli or FSGS with or without mesangial proliferation, (3) RB findings of diffuse and severe podocyte effacement on electron microscopy, and (4) the absence of subendothelial/subepithelial immune deposits on LM, IF, and EM, or their sole confinement to the mesangium [8]. Our patient fulfilled the 2019 EULAR/ACR classification criteria for SLE (ANA, autoimmune hemolysis, proteinuria, and decreased C4 associated with RB showing a histological pattern of tip lesion variant FSGS), with thickening of the glomerular basement membrane and no mesangial proliferation. In the Hu et al. study [9], out of the 3750 patients with SLE that were analyzed, 1.33% of the cases were identified as LP, of which 18% showed FSGS lesions. Lopez et al. reported a case series of eight patients with LP, of which only one showed FSGS lesions [18]. However, Batlle et al. reported a case series of four patients with LP, where all patients showed FSGS lesions [19]. Primary FSGS is differential in our case due to the finding in the LM and the presence of complete criteria for nephrotic syndrome. However, this possibility is ruled out since the patient has EULAR/ACR criteria for SLE. The absence of endocapillary proliferation or crescents in the LM distances the option of proliferative glomerulonephritis. On the one hand, Hu et al. [9] found that LP presents as glomerular deposition confined to the mesangium or without deposition. Most patients had both immunoglobulin and complement deposits in the glomeruli (86%), and only 3% had no deposits at all. The DIF of our patient showed the absence of any immune deposits, as reported in the minority of cases. In addition, Hu et al. [9] reported that almost 90% of RBs had pedicle effacement ≥ 70% and subendothelial or subepithelial deposits were virtually absent. In our patient, EM showed pedicellar effacement in 70% of the sample and the absence of subepithelial/endothelial deposits. A pedicellar effacement of less than 80% corroborates secondary FSGS; furthermore, the absence of immune deposits rules out the differential options of class III, IV (subendothelial deposits), and class V (subepithelial deposits). Furthermore, EM showed a diffuse thickening of the GBM which is not characteristic of LP, but rather, supported by evidence of diabetic retinopathy, appears to be an early structural change, which is evident within 1.5 to 2 years of diagnosis of T1DM [20], so it would be an overlapping condition.

Regarding predictors of response to treatment, the histological subtype is one key predictor of treatment response in LP. Hunt et al. reported that FSGS forms have a worse response to steroids than MCD. The FSGS subtype had a low rate of complete remission (22.2%), and most patients had a partial remission (55.6%) or non-response (22.2%) [9]. However, there are differences in the clinical characteristics and outcomes of FSGS patients according to these pathologic variants. In general, the collapsing variant has been reported to have a worse renal survival rate compared to other variants, while the tip variant shows the best prognosis and high rates of complete remission, as in our case [21]. Additionally, the degree of podocyte foot process effacement observed in EM (≥70%) is a valuable predictor, distinguishing LP from other forms of LN [22]. Another predictor is the absence of subendothelial or subepithelial immune deposits, supporting the effectiveness of immunosuppressive therapy, particularly when implemented early in the disease course [9]. In contrast, incomplete responses in cases of collapsing FSGS pose a significant challenge, with an increased risk of progression to end-stage renal disease if not adequately managed [5]. Furthermore, LP usually presents with nephrotic syndrome, which presents complications that are well described in the literature. Another challenge is the risk of adverse effects associated with immunosuppressive therapy. While corticosteroids remain effective, their prolonged use is linked to metabolic complications and infection risks.

Regarding management, there are currently no randomized clinical trials on LP. The Spanish Group for the Study of the Glomerular Diseases (GLOSEN) guidelines recommend treatment like that for minimal lesion nephrotic syndrome, including corticosteroids as the first line of treatment, with calcineurin inhibitors as the second line in corticosteroid-resistant patients [23]. The Kidney Disease Improving Global Outcomes (KDIGO) 2024 guideline suggests considering combined maintenance therapy with low-dose glucocorticoids and another immunosuppressive agent [24]. Hu et al. [9] demonstrated that steroids alone or in combination with another immunosuppressive agent induced remission in 94% of cases. More than half of PL patients relapse, and this often coincides with the extrarenal manifestations and/or serological activity of SLE [9]. In the Lopez et al. study, all patients received medical therapy either with steroids, mycophenolate mofetil (MMF), and/or hydroxychloroquine. All the patients treated achieved partial renal response [18]. In the Batlle et al. study, all patients did not respond to monotherapy with glucocorticoids, requiring other immunosuppressive agents [19]. Our patient was treated with steroids, mycophenolate mofetil (MMF), and hydroxychloroquine (HQC) in addition to antiproteinuric drugs (losartan and spironolactone), obtaining a complete response to the nephrotic syndrome. This result is like the one described in the literature because despite presenting an FSGS-type lesion, it was a TIP type, which has the best prognosis.

The Phase 3 Advancing Understanding of RecOvery afteR traumA (AURORA) 2 clinical trial showed, in a treatment with a follow-up of 3 years, that voclosporin in combination with MMF and low-dose steroids had a clinically and statistically better renal response rate than MMF and low-dose steroids alone in patients with LN [25]. However, this study did not include patients with LP; therefore, clinical trials are required to assess the efficacy of different treatment options in conjunction with steroids.

## 4. Conclusions

To conclude, we describe the clinical, analytical, and histological manifestations of an unusual case of LP in a 28-year-old woman with T1DM and newly diagnosed SLE. LP is a rare disease that is a separate entity from the six ISN/RPS classes whose pathophysiology is not fully understood yet. Renal biopsy is a key diagnostic tool in this case, which showed FSGS. The complete remission of nephrotic syndrome in this patient after the treatment of mycophenolate mofetil (MMF), hydroxychloroquine (HQC), and steroids reinforces the need for personalized treatments based on the histological characteristics of the patient. Despite being a rare disease, the early recognition of LP can significantly improve the renal and overall prognosis of patients. This report is the first case of LP described in Peru.

## Figures and Tables

**Table 1:** Laboratory findings on admission.

	Values	Normal Range United
Hemoglobin (Hb)	6.8	11–15 g/dL
Reticulocytes	21.4%	0.5–2%
Platelet count	266	150–400 × 10^3^ µL
White blood cells (WBC)	13.7	5–10 × 10^3^ µL
Limphocytes	3.1	1.5–3.5 × 10^3^ µL
Lactate dehydrogenase (LDH)	244	120–246 U/L
Direct Coombs test	+++	NA
Total bilirrubin	1.3	0.2–1.3 mg/dL
Indirect bilirrubin	0.2	0–1.1 mg/dL
Total proteins	5.5	6.3–8.2 g/dL
Serum albumin	2.3	3.5–5 g/dL
Glucose	305	75–110 mg/dL
Urea	16	15–36 mg/dL
Creatinine	0.37	0.7–1.2 mg/dL
Sodium	131	135–148 mmol/L
Potassium	3.7	3.5–5.3 mmol/L
Total calcium	8.2	8.4–10.2 mg/dL
Phosphorus	4.9	2.5–4.5 mg/dL
Magnesium	1.7	1.6–2.3 mg/dL
Total cholesterol	362	0–200 mg/dL
Triglycerides	176	<150 mg/dL
LDL	244.8	0–130 mg/dL
AST	28	15–46 U/L
ALT	23	13–69 U/L
ESR	140	0–15 mm/h
CRP	Negative	mg/L
Hematuria	0–2 xc	NA
Anti-VHB core	Not Reactive	NA
HBsAg	Not Reactive	NA
Anti-VHC	Not Reactive	NA
Anti-VIH 1-2	Not Reactive	NA
RPR	Not Reactive	NA
Anti-HTLV 1-2	Not Reactive	NA
Bengal rose test	Negative	NA
Blood culture	Negative	NA
Urine culture	Negative	NA
Parasitolgy stool	Negative	NA
24 h urine total protein Excretion	15,640	42–225 mg/24 h
ANA	1/800	NA
Anti-dsDNA		
C3	103	90–180 mg/dL
C4	9	10–40 mg/dL

NA: Not applicable, LDL: Low-Density Lipoprotein, AST: Aspartate Aminotransferase, ALT: Alanine Aminotransferase, ERS: Erythrocyte Sedimentation Rate, CPR: C-reactive protein, Anti-VHB core: Anti-Hepatitis B Core Antibody, HBsAg: Hepatitis B Surface Antigen, Anti-VHC: Anti-Hepatitis C Antibody, Anti-VIH 1-2: VIH 1-2 Antibody, RPR: Rapid Plasma Reagin, Anti-HTLV1-2: Human T-Lymphotropic Virus 1-2 Antibody, ANA: Antinuclear Antibody, anti-dsDNA: C3: Complement C3, C4: Complement C4.

## Data Availability

No new data were created or analyzed in this study. The data used to support the findings of this study are available from the corresponding author on request (contact J.C.D.L.F., jflomer@mde.es).

## References

[1] Aringer M., Costenbader K., Daikh D., Brinks R., Mosca M., Ramsey-Goldman R., Smolen J.S., Wofsy D., Boumpas D.T., Kamen D.L. (2019). 2019 European League Against Rheumatism/American College of Rheumatology Classification Criteria for Systemic Lupus Erythematosus. Arthritis Rheumatol..

[2] Ugarte-Gil M.F., Alarcón G.S. (2023). Systemic lupus erythematosus in Latin America: Outcomes and therapeutic challenges. Clin. Immunol. Commun..

[3] Parikh S.V., Almaani S., Brodsky S., Rovin B.H. (2020). Update on Lupus Nephritis: Core Curriculum 2020. Am. J. Kidney Dis..

[4] Weening J.J., D’agati V.D., Schwartz M.M., Seshan S.V., Alpers C.E., Appel G.B., Balow J.E., Bruijn J.A.N.A., Cook T., Ferrario F. (2004). The classification of glomerulonephritis in systemic lupus erythematosus revisited. Kidney Int..

[5] Oliva-Damaso N., Payan J., Oliva-Damaso E., Pereda T., Bomback A.S. (2019). Lupus Podocytopathy: An Overview. Adv. Chronic Kidney Dis..

[6] Hoover P.J., Costenbader K.H. (2016). Insights into the epidemiology and management of lupus nephritis from the US rheumatologist’s perspective. Kidney Int..

[7] Lewis E.J., Lewis E.J., Schwartz M.M., Korbet S.M., Chan D.T.M. (2010). Lupus Podocytopathy. Lupus Nephritis.

[8] Bomback A.S., Markowitz G.S. (2016). Lupus Podocytopathy: A Distinct Entity. Clin. J. Am. Soc. Nephrol..

[9] Hu W., Chen Y., Wang S., Chen H., Liu Z., Zeng C., Zhang H., Liu Z. (2016). Clinical-Morphological Features and Outcomes of Lupus Podocytopathy. Clin. J. Am. Soc. Nephrol..

[10] Pipino A.S., Segretti P.M., Ribeiro B.S., Gomes G.M., de Segura J.A., Giovanni Filho S.C.F., Zanuto A.C.D. (2024). WCN24-2512 Lupus podocytopathy: Case report. Kidney Int. Rep..

[11] Kraft S.W., Schwartz M.M., Korbet S.M., Lewis E.J. (2005). Glomerular podocytopathy in patients with systemic lupus erythematosus. J. Am. Soc. Nephrol..

[12] Forbes J.M., Cooper M.E. (2013). Mechanisms of diabetic complications. Physiol. Rev..

[13] Hünemörder S., Treder J., Ahrens S., Schumacher V., Paust H., Menter T., Matthys P., Kamradt T., Meyer-Schwesinger C., Panzer U. (2015). T_H1_ and T_H17_ cells promote crescent formation in experimental autoimmune glomerulonephritis. J. Pathol..

[14] Paquissi F.C., Abensur H. (2021). The Th17/IL-17 axis and kidney diseases, with focus on lupus nephritis. Front. Med. Nephrol..

[15] Wang Y., Yu F., Song D., Wang S.-X., Zhao M.-H. (2014). Podocyte involvement in lupus nephritis based on the 2003 ISN/RPS system: A large cohort study from a single centre. Rheumatology.

[16] Alqudsi M., Cobbs J., Navarrete J. (2019). Lupus Podocytopathy Case Series in an Urban USA Population. Am. J. Kidney Dis..

[17] Salvatore S.P., Barisoni L.M.C., Herzenberg A.M., Chander P.N., Nickeleit V., Seshan S.V. (2012). Collapsing glomerulopathy in 19 patients with systemic lupus erythematosus or lupus-like disease. Clin. J. Am. Soc. Nephrol..

[18] Vasquez D.L., Bembry W., Mesa C.J., Patel N.J., Guevara M.E. (2022). Lupus podocytopathy: Case series and review. Lupus.

[19] Dina-Batlle L., Rodriguez D.A., Bencosme E. (2024). Lupus podocytopathy: Case series of 4 cases from Dominican Republic. Kidney Int. Rep..

[20] Alicic R.Z., Rooney M.T., Tuttle K.R. (2017). Diabetic Kidney Disease: Challenges, Progress, and Possibilities. Clin. J. Am. Soc. Nephrol..

[21] Thomas D., Franceschini N., Hogan S., Holder S.T., Jennette C., Falk R., Jennette J. (2006). Clinical and pathologic characteristics of focal segmental glomerulosclerosis pathologic variants. Kidney Int..

[22] Dube G.K., Markowitz G.S., Radhakrishnan J., Appel G.B., D’Agati V.D. (2002). Minimal change disease in systemic lupus erythematosus. Clin. Nephrol..

[23] Rojas-Rivera J.E., García-Carro C., Ávila A.I., Espino M., Espinosa M., Fernández-Juárez G., Fulladosa X., Goicoechea M., Macía M., Morales E. (2023). Consensus document of the Spanish Group for the Study of the Glomerular Diseases (GLOSEN) for the diagnosis and treatment of lupus nephritis. Nefrologia Engl. Ed..

[24] Rovin B.H., Ayoub I.M., Chan T.M., Liu Z.H., Mejía-Vilet J.M., Floege J. (2024). Kidney Disease: Improving Global Outcomes (KDIGO) Lupus Nephritis Work Group. KDIGO 2024 Clinical Practice Guideline for the management of LUPUS NEPHRITIS. Kidney Int..

[25] Saxena A., Ginzler E.M., Gibson K., Satirapoj B., Santillán A.E.Z., Levchenko O., Navarra S., Atsumi T., Yasuda S., Chavez-Perez N.N. (2024). Safety and efficacy of long-term voclosporin treatment for lupus nephritis in the phase 3 AURORA 2 clinical trial. Arthritis Rheumatol..

